# Flexible Strain Sensors Based on Printing Technology: Conductive Inks, Substrates, Printability, and Applications

**DOI:** 10.3390/ma18092113

**Published:** 2025-05-04

**Authors:** Xue Qi, Jingjing Luo, Haipeng Liu, Shuheng Fan, Zhongqi Ren, Peike Wang, Suzhu Yu, Jun Wei

**Affiliations:** 1 Shenzhen Key Laboratory of Flexible Printed Electronics Technology, Harbin Institute of Technology (Shenzhen), Shenzhen 518055, China; 2 School of Materials Science and Engineering, Harbin Institute of Technology (Shenzhen), Shenzhen 518055, China; 3 State Key Laboratory of Advanced Welding and Joining, Harbin Institute of Technology (Shenzhen), Shenzhen 518055, China

**Keywords:** flexible strain sensor, printing technology, conductive ink development, printability, flexible electronics applications

## Abstract

Printing technology has revolutionized manufacturing by enabling high-volume, multipurpose, low-cost production with minimal environmental impact. This advancement has led to the integration of flexible electronic devices, such as displays, actuators, and sensors, into various consumer markets. Over the past few decades, printed electronics have garnered significant interest from both academic and industrial communities. Among these, flexible strain sensors stand out due to their adaptability and potential for large-scale applications. However, a comprehensive analysis of their sensing performance, particularly with respect to printability, remains lacking. This review aims to systematically explore the development of flexible strain sensors produced through printing technology, focusing on key aspects such as the formulation of conductive inks, the use of diverse substrate materials, and the challenges associated with printability. Additionally, it delves into the practical applications of these sensors across multiple industries. By providing an in-depth examination of these factors, this review offers valuable insights into the current state of printed electronics and highlights their future potential in advancing flexible sensing technologies.

## 1. Introduction

As a cornerstone of modern information technology, sensors play a crucial role in various fields, ranging from industrial automation and healthcare to environmental monitoring and consumer electronics [1,2]. A sensor is broadly defined as a detection device that senses a physical, chemical, or biological parameter and converts this information into a readable or transmittable signal [3]. This conversion is often achieved through electrical signals that can be displayed, recorded, or further processed for various applications [4]. By utilizing physical, chemical, and biological interactions, sensors transform non-electrical quantities, such as temperature, pressure, and chemical composition, into electrical outputs [5].

Sensors generally consist of three main components: a sensitive element, a conversion element, and conversion circuits [6]. The sensitive element directly interacts with the target environment, detecting changes in physical, chemical, or biological states. The conversion element then processes these changes into electrical signals suitable for further transmission or measurement [7]. This process enables sensors to extend beyond the limitations of human senses, allowing us to detect conditions or materials that our natural senses cannot perceive, such as toxic gases or microscopic biological activities [8,9]. With the development of advanced sensors, the scope and accuracy of data acquisition have expanded significantly, enabling real-time monitoring of complex processes and environments [10].

Despite their diverse applications, sensors face ongoing challenges related to power consumption, miniaturization, cost-efficiency, and lifespan. Addressing these challenges is key to unlocking the potential of next-generation sensors. Flexible strain sensors, which measure mechanical deformation by converting strain into electrical signals, have emerged as a critical area of research. These sensors are particularly relevant in applications where conventional rigid sensors are not suitable, such as in wearable devices, soft robotics, and electronic skins [11]. They can detect a wide range of deformations, making them ideal for monitoring physiological activities and structural health in a non-invasive manner.

The lack of a unified classification system for sensors reflects the breadth of their design and application. Common classification methods include categorization based on working principles—such as resistive, capacitive, piezoelectric, or photoelectric sensors—or based on their application areas, such as pressure, temperature, or displacement sensors [12,13]. For flexible strain sensors specifically, the choice of materials and fabrication methods plays a crucial role in determining their sensitivity, durability, and application scope. These sensors can be developed using diverse techniques, but recent advancements in printing technology have opened new avenues for creating high-performance, cost-effective, and customizable sensors.

**Figure 1:**
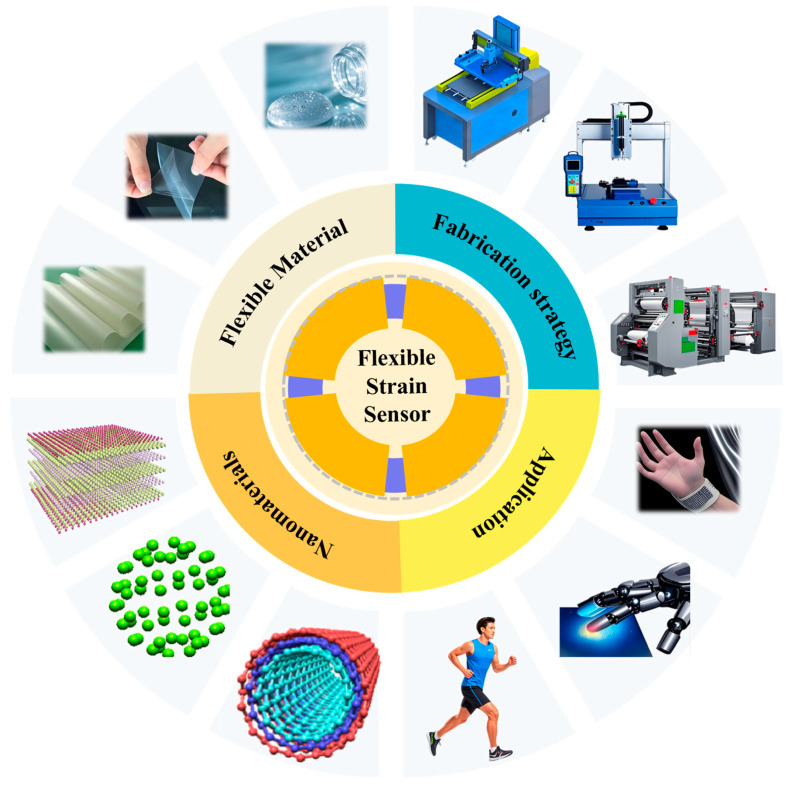
An overview of flexible strain sensors, encompassing flexible materials, fabrication strategies, application scenarios, and operational mechanisms.

Printing technology has enabled the development of flexible strain sensors with greater scalability and material versatility. Unlike traditional fabrication methods, printing allows for the rapid deposition of conductive materials onto various flexible substrates, paving the way for lightweight and adaptable sensor designs (Figure 1). This review is dedicated to a comprehensive and systematic exploration of printed flexible strain sensors.

## 2. Sensor Performance Characteristics

### 2.1. Sensor Static Characteristics

The static properties of the sensor are expressed in terms of input and output relationships without time variables. The following parameters (linearity, sensitivity, hysteresis, repeatability, etc.) can be used to characterize its static characteristics [14,15,16].

Linearity: The ideal input–output feature of a sensor should be linear. Linearity is based on a specific fitted straight line as a benchmark and compared with the calibration curve. The maximum deviation of the inconsistency is calculated as a percentage of the theoretical full-scale output value:

$$\delta_{L}=\pm \frac{\left|\Delta L_{max}\right|}{Y_{FS}}\times 100\%$$

*Y_FS_* = *y_max_* − *y_min_*—full-scale output voltage [17,18].

Sensitivity: The ratio of the change Δ*y* of the sensor output to the input change Δ*x* that causes the difference is its static sensitivity [17,19], and its expression is

k=Δy/Δx

Hysteresis: The hysteresis characteristic describes how the sensor loading (increasing input) and unloading (decreasing intake) input–output characteristic curves do not coincide. Experimental methods generally measure it [20].

Repeatability: Repeatability refers to the degree of inconsistency in the characteristic curve obtained when the sensor input is continuously changed over the entire range in the same direction many times [21].

Resolution: The output changes observably after a specific increment when the sensor input increases slowly from a non-zero value. This input increment is called the sensor’s resolution, the minimum input increment [18].

### 2.2. Sensor Dynamics Characteristics

The dynamic characteristics of flexible strain sensors are critical in evaluating their performance, particularly in applications where real-time monitoring and adaptability are necessary. Key parameters such as response time, frequency response, and dynamic range determine how well a sensor can detect and measure strain in dynamic environments. The dynamic characteristics of a sensor refer to the response (output) parts of the sensor to excitation (input) [18].

#### 2.2.1. Response Time

The response time of a flexible strain sensor refers to the duration it takes for the sensor to react to a change in strain and produce a stable output signal. It is a crucial parameter for applications where real-time strain measurement is required, such as wearable health monitoring and soft robotics. In these contexts, a shorter response time enables the sensor to track rapid changes in movement or deformation accurately. For instance, in sports performance tracking, sensors with a fast response time can quickly capture dynamic body movements, providing immediate feedback for athletes. Conversely, a slower response time may result in delayed feedback, which can reduce the effectiveness of the sensor in applications that require immediate data interpretation [22].

#### 2.2.2. Frequency Response

The frequency response characterizes the sensor’s ability to accurately measure strain variations across different frequencies [23]. This parameter is essential in determining the sensor’s bandwidth, indicating the range of frequencies over which the sensor operates effectively. A broader frequency range allows a sensor to detect both low-frequency movements, such as gradual bending, and high-frequency vibrations, such as mechanical shocks or impacts. This makes a wide frequency response particularly valuable in structural health monitoring, where a sensor needs to detect small vibrations as well as sudden structural changes. In wearable devices, a wide frequency range ensures accurate tracking of diverse activities, from slow walking to fast running or jumping. Sensors with a limited frequency response may fail to capture rapid changes, leading to incomplete data in dynamic environments.

#### 2.2.3. Dynamic Range

The dynamic range of a flexible strain sensor is defined as the ratio between the smallest and largest strain levels that the sensor can accurately detect. It is a critical parameter for applications requiring the measurement of both minor deformations and substantial changes. A wide dynamic range enables the sensor to capture small, precise strain changes in applications like electronic skins for robotics, as well as larger deformations in flexible displays. For example, a flexible strain sensor used for monitoring physiological signals like breathing might need to detect both subtle chest movements and larger, more pronounced breathing patterns. A sensor with a narrow dynamic range might struggle to accurately measure the full range of deformations, either missing small signals or saturating when detecting larger deformations [24].

These dynamic characteristics—response time, frequency response, and dynamic range—determine the versatility and suitability of flexible strain sensors for different applications. Each characteristic must be optimized according to the specific needs of the application to ensure accurate, real-time monitoring. For example, while wearable sensors benefit from short response times and broad frequency ranges, structural monitoring sensors may prioritize a wide dynamic range. Understanding and optimizing these characteristics is key to developing high-performance flexible strain sensors that can meet the requirements of diverse and rapidly evolving technological fields.

## 3. Printed Sensors and Printing Techniques

Sensors have become a vital component in the digital age, playing a crucial role in turning complex and often inaccessible data into easy-to-interpret, actionable information. From smartphones and intelligent voice devices to industrial equipment and energy platforms, sensors act as bridges between humans and the natural world [25]. As sensor technology has evolved, it has been coupled with advancements in data storage, energy storage, materials science, and network infrastructure. These developments, combined with the decreasing costs of production, have led to an expanding range of sensor applications. One such innovation is the use of printed sensors, a technology that has revolutionized sensor production in recent years [26,27].

Traditional sensors, typically made from rigid materials like silicon, ceramics, and glass, have inherent limitations, such as high production costs, limited flexibility, and shorter lifespans [28]. While these sensors remain indispensable, they lack versatility in terms of volume, cost-effectiveness, and long-term performance. In contrast, printed sensors, which are often fabricated using polymers and specialized conductive inks, leverage flexible substrates such as plastic, paper, or textiles. The use of various printing techniques allows these sensors to achieve a wide range of benefits, including large-area coverage, high responsiveness, and significant cost reductions, offering unique advantages over traditional sensor technologies [28,29].

A key enabler of printed sensor technology is the diversity of printing techniques, each offering unique advantages in terms of resolution, material compatibility, and scalability. These techniques can be broadly categorized into template-based (contact) printing, digital (non-contact) printing, and other advanced patterning methods.

Template-based printing methods include screen printing [30], gravure printing [31], flexographic printing [32], and offset printing [33]. These methods use physical masks or patterned surfaces to transfer ink onto substrates and are generally well-suited for high-throughput production. They offer good control over layer thickness and are compatible with a wide range of functional inks. However, their resolution is typically limited to the scale of tens to hundreds of micrometers.

In contrast, digital printing techniques, such as inkjet printing [34], aerosol jet printing [35], and electrohydrodynamic (EHD) printing [36], are non-contact and do not require physical masks or plates. These methods can deposit materials with high precision and are ideal for rapid prototyping and patterning complex geometries. Inkjet printing, for instance, allows for resolutions down to 20–50 µm and is particularly advantageous for printing on flexible substrates.

The choice of printing technique significantly influences the electrical, mechanical, and functional performance of printed sensors. Factors such as ink rheology, curing conditions, and substrate interaction vary across methods, and selecting the appropriate approach is crucial to achieving the desired performance. As these technologies continue to evolve, printed sensors are expected to find broader adoption in applications ranging from healthcare and soft robotics to structural monitoring and wearable electronics.

### 3.1. Inkjet Printing

Inkjet printing is a non-contact, pressure-free, plate-free printing. Without a physical mask or an image carrier, direct deposition techniques are applied that allow in-computer digital image signals to print designs by propelling ink droplets onto the substrate [29].

**Figure 2:**
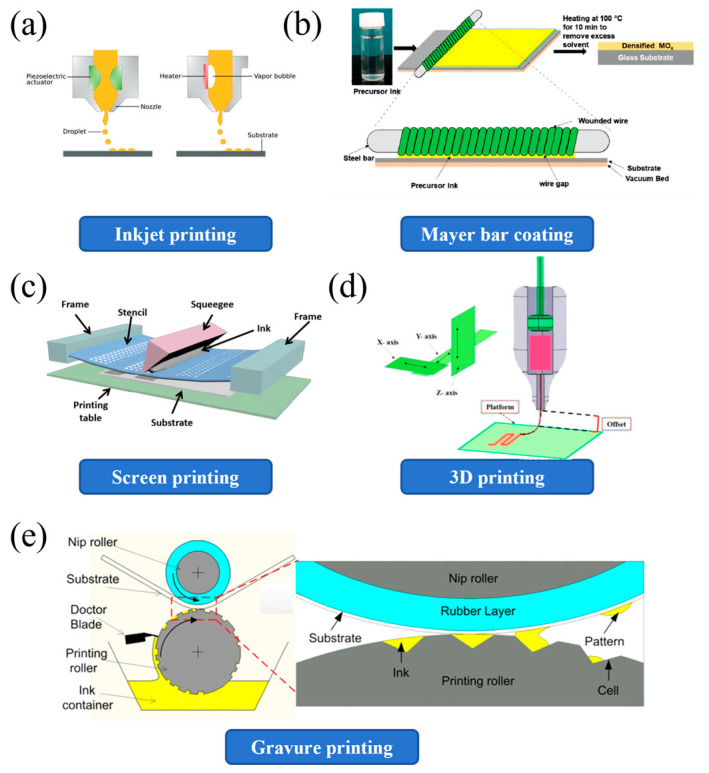
Key printing and coating techniques: This figure illustrates various printing and coating methods used in manufacturing. (**a**) Inkjet printing is shown with both continuous and on-demand systems, highlighting the droplet ejection process [68]. Copyright © 2020 The Royal Society of Chemistry. (**b**) Mayer Bar Coating depicts the application of a wet film onto a substrate using a bar, followed by drying [69]. Copyright © 2020 by the authors. Licensee MDPI, Basel, Switzerland. (**c**) Screen Printing demonstrates the use of a squeegee to push ink through a stencil onto a substrate [70]. Copyright © 2022 by the authors. Licensee MDPI, Basel, Switzerland. (**d**) Three-dimensional printing illustrates the layer-by-layer deposition process, with movement along the X, Y, and Z axes [71]. Copyright © 2020 by the authors. (**e**) Gravure printing shows the transfer of ink from a rotating cylinder to a substrate [72]. Copyright © 2013 IOP Publishing Ltd. These techniques are essential for producing high-quality prints and coatings across different industries.

As shown in Figure 2a, inkjet is divided into continuous inkjet and drop-on-demand inkjet according to the different ink transfer methods. In constant inkjet printing, the working principle is that a voltage source electronically controls the continuous flow of ink droplets. Part of the droplets is electrostatically charged and deflected by electrostatic deflectors for negative printing, while the uncharged droplets are used to print the desired image onto the substrate. In contrast, drop-on-demand inkjet printing is based on the principle that droplets are formed only when the image signal requires it to obtain the desired print. On-demand inkjet printing is refined into thermal inkjet and piezoelectric inkjet printing processes and has the advantages of maskless manufacturing, high printing resolution, and low cost [37,38,39].

### 3.2. Bar Coating

Mayer Rod is one of the main popular coating methods. The Mayer Rod is a stainless steel rod tightly wound with stainless steel wires of different diameters. Put the excess solution on the substrate and use a stick to spread the answer on the substrate (Figure 2b). This device operates by scraping off excess coating solution and regulating the film thickness through the precise diameter of the wire used to wind the rod. The wet film thickness is determined by the gap between the wire and the substrate, which can be adjusted to accommodate a range of desired coating thicknesses. Rods are available in various wire diameters to provide flexibility in coating applications. It is important to note that the dry film thickness is ultimately determined by the solid concentration of the coating solution, which influences the final thickness after solvent evaporation [40,41].

The scalability and simplicity of this technology render it highly appealing to both research and manufacturing communities. Moreover, its adaptability facilitates its integration into various experimental setups, making it an ideal subject for study. This method shares several similarities with doctor blading techniques but offers enhanced simplicity and ease of repeatability, attributed to the use of a fixed coating thickness rod. Given its similarities in coating mechanisms [42,43,44].

### 3.3. Screen Printing

Screen printing is a popular and well-established technique for transferring paste-like materials (inks) onto substrates. Screen printing is usually performed by hand or semi- or fully automatic systems. Screen printers have a simple setup consisting of a squeegee, a stencil, and a screen with a design (Figure 2c) [45,46]. Printing is carried out using the fundamental principle that the mesh of the screen printing plate is transparent to the ink, and the mesh of the non-graphic part is impermeable to the ink [47]. When printing, pour ink on one end of the screen printing plate, apply a certain pressure to the ink part of the screen printing plate with a scraper, and move to the other end of the screen printing plate at the same time [48]. The ink is squeezed onto the substrate by the scraper from the mesh of the graphic part during the movement. Due to the viscosity of the ink, the imprint is fixed within a specific range. During the printing process, the squeegee is always in line contact with the screen printing plate and the substrate, and the contact line moves with the movement of the squeegee [49]. A particular gap is maintained between them so that the screen printing plate, during printing, generates a reaction force to the squeegee through its tension. This reaction force is called rebound force [50]. Due to resilience, the screen printing plate and the substrate are only in moving line contact, while other parts of the screen printing plate and the substrate are separated. The ink and the screen are broken, ensuring printing dimensional accuracy and avoiding smudging the substrate. When the squeegee scrapes the entire layout and lifts, the screen printing plate is also lifted, and the ink is gently rubbed back to the original position. So far, it is a printing trip [51].

### 3.4. Flexographic Printing

Flexographic printing is a method of printing using a flexographic printing plate and transferring ink through an anilox roller. It is a type of letterpress printing process called flexo printing [52]. The embossing cylinder, plate, anilox roll, doctor blade, and inking unit are the main components of flexographic printing. The graphic part of the flexographic printing plate is raised. The anilox roller evenly coats the ink layer of a certain thickness on the graphic part of the printing plate during printing. Then, under the pressure of the embossing roller, the ink layer of the graphic part is transferred to the surface of the substrate to form high-resolution graphics [53]. The inking mechanism of a flexographic printing press is usually a two-roller inking mechanism, and R2R high-throughput rotary printing has high-speed printing capabilities [46,53,54]. Flexographic printing is the use of a flexographic plate through the anilox roller to transfer ink printing [55]. It requires a high elastic letterpress press and a perforated total anilox roll for quantitative ink supply, and the printing ink has good fluidity and viscosity. With lower fast-drying solvent or water-based inks, the print quality is comparable to litho. It is suitable for printing various printing materials such as paper, plastic film, metal film, self-adhesive, etc. Flexographic printing machine, because of the metal anilox ink supply system with a short ink path, the amount of ink is easy to control, and the automation program is high. Therefore, the operation technology of printing is simpler than letterpress printing and offset printing [56].

### 3.5. Three-Dimensional Printing

Three-dimensional printing, also known as 3D printing, additive manufacturing (English: additive manufacturing, AM), and laminate manufacturing, can refer to any process of three-dimensional printing objects (Figure 2d). Three-dimensional printing is primarily additive, layering raw materials under computer control [57,58]. The content of 3D printing can be derived from 3D models or other electronic data, and the printed 3D objects can have any shape and geometric features [59,60].

The original meaning of the term “3D printing” refers to the process of orderly depositing materials, including thermoplastics such as acrylonitrile butadiene styrene, metals, resins, and ceramics, into a powder layer through an inkjet printhead [61]. More recently, the definition of the term has been expanded to encompass a wide variety of technologies, such as extrusion and sintering processes. Technical standards generally use “additive manufacturing” to convey this broad meaning [62].

### 3.6. Gravure Printing

Gravure printing is an impact-based printing technique. The image carrier cylinder (gravure cylinder), doctor blade, ink reservoir, and die cylinder are some of the main components of a gravure printing press (Figure 2e). It directly imprints the ink contained in the gravure micro-units on the substrate. The gravure-printed printing plate is composed of micro-units, also called small cells, corresponding to the original graphics and text and the surface of the printing plate. Microcells are engraved by using electromechanical means or using lasers [63,64,65]. The small cells fill with ink as the gravure cylinder rotates in the ink fountain. The scraper wipes off excess ink remaining on the surface of the cylinder. The angle of the squeegee also plays a key role in printing [66]. When the ink enters between the engraving cylinder and the impression cylinder, the ink is transferred to the rollable substrate by capillary action. The surface properties of the substrates are also modified to facilitate ink transfer from the cells. Solution properties and cell width/depth ratio play important roles in gravure printing and other system parameters [63]. Gravure printing has significant advantages in high-resolution printing, high speed, low viscosity inks, and a simple ink transfer [67].

## 4. Printed Electronics Material

Conductive inks serve as the foundational elements in flexible printed strain sensors, enabling precise device fabrication through high-resolution patterning while maintaining compatibility with deformable substrates. These functional materials are systematically classified into four categories based on their conductive filler systems: metal-based, carbon-based, polymer-based, and composite-based architectures.

Metal-based systems, exemplified by silver nanoparticles and liquid metal alloys, deliver ultrahigh conductivity critical for sensitive strain detection. Carbon-based variants utilizing graphene or carbon nanotubes achieve balanced performance through conjugated molecular networks, offering tunable flexibility and cost-effective manufacturing. Polymer-driven formulations leverage molecular chain reconfiguration mechanisms to achieve extreme stretchability, while composite hybrids synergistically combine metallic, carbon, or polymeric components to overcome individual material limitations.

This strategic categorization allows for the optimization of key parameters, including sensitivity, strain range, and hysteresis, across diverse operational scenarios. The printability of these inks facilitates scalable production of conformal sensors, driving innovations in personalized healthcare monitoring and adaptive human–machine interfaces [73,74]

### 4.1. Metal Inks

**Figure 3:**
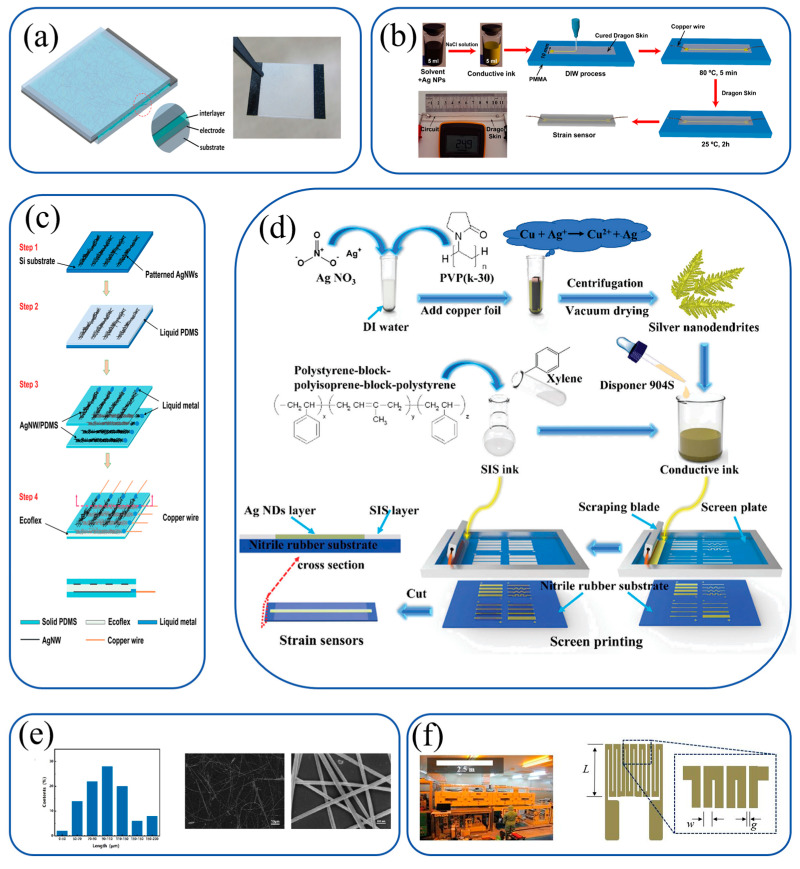
Metal-based inks for flexible sensor applications. (**a**) Wang et al.: dielectric enhancement from AgNWs embedded in PU dielectric modulates capacitance changes under compression for pressure sensing [75]. Copyright © 2015 The Royal Society of Chemistry. (**b**) Huang et al.: NaCl-induced sintering of Ag NPs in 3D-printable ink creates conductive aggregates stabilized by hydrogen bonds, enabling crack-free stretchability via solvent-mediated aggregate mobility [76]. Copyright © 2021 American Chemical Society. (**c**) Yao et al.: capacitive coupling between AgNW conductors and Ecoflex/PDMS dielectrics enables multi-modal strain/pressure/touch detection through capacitance variations [77]. Copyright © 2014 The Royal Society of Chemistry. (**d**) Tian et al.: microcrack propagation in screen-printed Ag nanodendrite films during stretching modulates electrical resistance proportional to strain [78]. Copyright © 2018 The Royal Society of Chemistry. (**e**) Qi et al.: piezoresistive effects from AgNW junction deformation in PDMS microstructures enable strain sensing via resistance changes [79]. Copyright © 2024 Wiley-VCH GmbH. (**f**) Park et al.: serpentine Ag patterns printed via roll-to-roll gravure accommodate mechanical strain through geometric deformation, maintaining conductivity [80]. Copyright © 2018 IOP Publishing Ltd.

In the dynamic field of flexible sensor engineering, silver-based metallic inks have emerged as a cornerstone due to their unique combination of electrical conductivity and mechanical compliance. A seminal contribution in this area is the work by Wang et al., who reported a breakthrough in pressure sensing through AgNW-filled polyurethane composites (Figure 3a). By screen-printing AgNW-laden aqueous ink into conductive electrodes and sandwiching them with PU dielectrics (0.1–3.0 wt% AgNW), the team achieved a permittivity enhancement from εr = 45.3 (3 wt%) to εr = 97.4 under compression. This enabled an unprecedented sensitivity of 5.54 kPa^−1^ for low pressures (<30 Pa) while maintaining stability over 200 bending cycles. Significantly, this system eliminated complex microfabrication steps, making it suitable for adhesive bandage integration—capable of detecting both direct motion and airflow-induced pressure changes—a testament to its clinical and industrial potential.

Building on this foundation, another innovative approach by Huang et al. leveraged 3D-printable Ag nanoparticle inks with liquid-metal-like properties (Figure 3b). By incorporating NaCl as a room-temperature sintering agent and stabilizing conductive aggregates via PAA-CMC hydrogen bonds, the ink demonstrated exceptional stretchability (800%) and cyclic durability (5000 cycles at 100% strain). The resulting sensors, with 248 S/cm conductivity and 83 ms response time, outperformed traditional rigid filler-based designs, particularly in maintaining conductive pathways under large deformations. This advance is particularly notable for its scalability, as the low-temperature (80 °C) sintering process aligns with roll-to-roll manufacturing requirements for next-generation wearable devices.

A complementary strategy was pursued by Yao et al., who integrated AgNWs with Ecoflex and PDMS to create multifunctional sensors (Figure 3c). The screen-printed AgNW electrodes provided both high conductivity and stretchability (50% strain), while the elastomeric matrix enabled conformal skin integration. This hybrid system achieved simultaneous detection of strain (GF = 0.7), pressure (1.62 MPa^−1^), and touch with sub-50 ms response times—a capability critical for applications in human–machine interfaces and healthcare monitoring. Importantly, the use of AgNWs minimized hysteresis, ensuring linear capacitance changes even under repeated loading.

For cost-effective and scalable solutions, Tian et al. introduced Ag nanodendrite inks synthesized via a facile copper-mediated reduction (Figure 3d). The dendritic microstructure (8–12 μm) conferred both high conductivity (1.14 × 10^5^ S/m) and printability, allowing tunable sensor performance through geometric engineering. By optimizing serpentine patterns, the team achieved sensitivities up to GF = 294.3 at 105% strain, combined with rapid response (18 ms) and long-term stability (>3 weeks). This work highlights the importance of microstructural design in balancing sensing performance and manufacturability, particularly for applications like gesture-recognition gloves.

A significant leap forward in AgNW synthesis was reported by Qi et al., who achieved record aspect ratios (1400) through a polyol method optimized with Br^−^/Cl^−^ ions and benzoin (Figure 3e). These ultra-long NWs (100 μm) with sub-100 nm diameters enabled highly conductive PDMS composites, which in turn supported microstructured sensors with GF = 322.2 at 5 μm feature sizes. Notably, these devices maintained linearity (R^2^ = 0.997) across 20–60% strain and exhibited no degradation after 10,000 cycles, setting new benchmarks for stretchable electronics.

Park et al. demonstrated the scalability of gravure-printed Ag sensors using roll-to-roll processing (Figure 3f). By optimizing ink formulation (1–10 μm particles, 16,570 mPa·s viscosity) and annealing conditions, the team achieved sensors with GF = 1.99–2.24 and strain ranges comparable to commercial devices (−386 × 10^−6^ to 16,272 × 10^−6^). While lower in sensitivity than composite-based designs, this method offers unmatched throughput (2 m/min) and resolution (30 μm), making it ideal for mass-producing strain sensors for industrial applications.

Collectively, these studies illustrate the versatility of silver-based inks in addressing critical challenges in flexible sensor development—from material innovation to scalable manufacturing. Each approach uniquely balances factors such as sensitivity, stretchability, and cost, underscoring the importance of tailoring ink properties to specific application requirements. Moving forward, further advancements in ink formulation and printing technologies will likely drive the integration of these sensors into next-generation smart systems.

### 4.2. Carbon Based Ink

**Figure 4:**
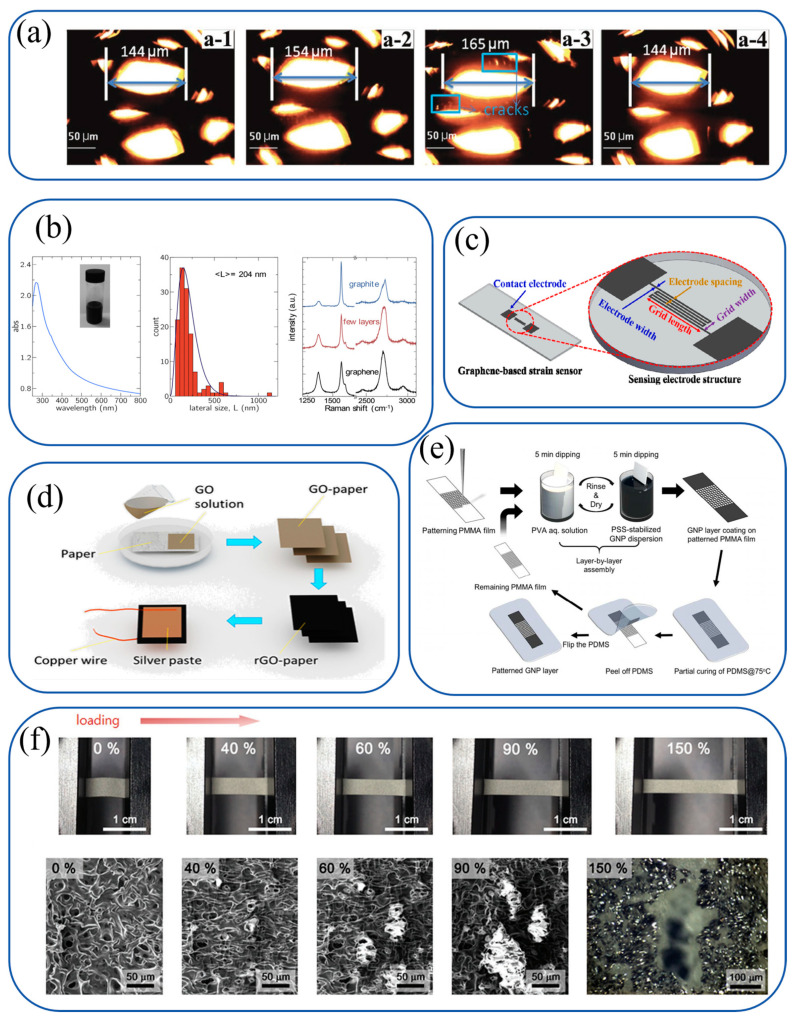
Carbon-based ink systems and their operational mechanisms for flexible sensors. (**a**) Li et al.: thin graphite foam (TGF) embedded in PDMS via low-temperature CVD. Operates through piezoresistive effects induced by crack generation in defective graphene layers under strain [81]. Copyright © 2017 WILEY-VCH Verlag GmbH & Co. KGaA. (**b**) Casiraghi et al.: water-based graphene ink with PS1 stabilizer for inkjet printing. Leverages porous ink properties to control drop spacing and layer thickness for tunable piezoresistivity [82]. Copyright © 2017 Elsevier Ltd. (**c**) Tseng et al.: screen-printed graphene sensors with ULDW-patterned grids. Relies on laser-induced microstructural changes to enhance piezoresponse under bending [83]. Copyright © 2021 Elsevier Ltd. and Techna Group S.r.l. (**d**) Tao et al.: rGO/tissue paper composites with porous structure. Exploits air gaps between layers for contact area modulation under pressure [84]. Copyright © 2017 American Chemical Society. (**e**) Lee et al.: layer-by-layer assembled GNP/PSS ink on PMMA templates. Achieves strain sensing via crack propagation in thin GNP layers constrained by micro-square patterns [85]. Copyright © 2017 American Chemical Society. (**f**) Yun et al.: RGO/PDMS composites via solution processing. Combines porous PDMS elasticity with RGO conductivity for piezoresistive strain sensing [86]. Copyright © 2017 WILEY-VCH Verlag GmbH & Co. KGaA.

Carbon-based inks, particularly graphene and its derivatives, have garnered significant attention in flexible sensor research due to their unique combination of electrical conductivity, mechanical flexibility, and biocompatibility. Herein, a comprehensive review of representative studies on carbon-based ink systems is presented, emphasizing their operational principles and technological advancements:

A scalable method for fabricating 3D graphite–polymer composite strain sensors was reported by Li et al., utilizing low-temperature CVD-grown thin graphite foam (TGF) embedded in PDMS (Figure 4a). The TGF, synthesized on nickel foam templates via ethylene CVD at 700 °C for 30 h, consists of ~60 graphene layers with high defect density. After nickel etching and PDMS infiltration, the composite exhibits piezoresistive behavior with a gauge factor (GF) of 52 at 100% strain. High defect density in thicker TGF layers promotes crack generation under strain, enhancing sensitivity compared to pristine graphene-based sensors.

Water-based and biocompatible graphene inks for inkjet-printed strain gauges on paper were developed by Casiraghi et al. Using ultrasonic-assisted liquid-phase exfoliation of graphite with 1-pyrenesulfonic acid sodium salt (PS1) stabilizer, the ink avoids toxic solvents and complex post-processing (Figure 4b). Graphene flakes (50–400 nm lateral size) printed on PEL P60 paper exhibit high conductivity and mechanical flexibility. Porous ink properties enable precise control over drop spacing and layer thickness, influencing sensor performance.

Highly sensitive screen-printed graphene strain sensors were fabricated by Tseng et al. via ultrafast laser direct writing (ULDW) for glass deformation detection (Figure 4c). Optimized ULDW parameters (31.9 J/cm^2^ fluence, 300 kHz frequency, 500 mm/s speed) created grid electrodes with varying lengths. The 6 mm grid sensor demonstrated superior performance, achieving a gauge factor of 550.14 and linear response (R^2^ = 0.9983) across 0–180 μm bending distances.

A novel graphene-paper pressure sensor incorporating reduced graphene oxide (rGO) on tissue paper was introduced by Tao et al. Tissue paper soaked in GO solution and thermally reduced at 250 °C for 5 h forms a porous, corrugated structure with air gaps between layers (Figure 4d). The eight-layer rGO sensor exhibits high sensitivity in the 0–2 kPa range, enabled by the tissue paper’s rough surface and interconnected pores.

Patterned graphene strain sensors were fabricated by Lee et al. using a water-based GNP/PSS dispersion and PVA solution (Figure 4e). Layer-by-layer assembly on CNC-milled PMMA templates allows precise control over GNP thickness (3–7 bilayers). Thinner layers (3 BLs) exhibit higher sensitivity due to enhanced crack propagation in micro-square patterns, enabling the detection of subtle motions.

Elastic graphene-based epidermal electronics were developed by Yun et al. via a low-cost solution-based approach (Figure 4f). Conductive reduced graphene oxide (RGO) films on porous PDMS (pPDMS) substrates were fabricated through steam-etching, electrostatic GO self-assembly, and hydriodic acid reduction. The RGO/pPDMS composites exhibit exceptional mechanical properties and biocompatibility, enabling seamless integration into wearable healthcare devices.

### 4.3. Polymer Inks

**Figure 5:**
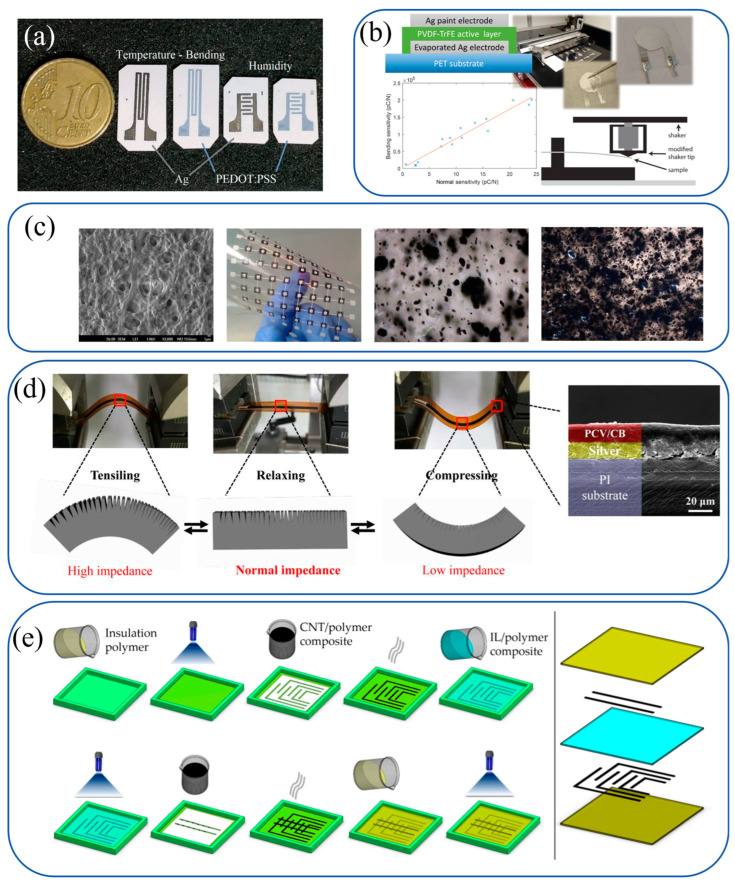
Polymer-based ink systems for flexible sensor applications. (**a**) Barmpakos et al.: inkjet-printed Ag-NP and PEDOT: PSS on paper. Ag-NPs enable stable humidity/temperature sensing via high conductivity, while PEDOT: PSS exhibits piezoresistive behavior [88]. Copyright © 2020 Elsevier B.V. (**b**) Rajala et al.: solution-processed P(VDF-TrFE) ink with vacuum-evaporated Ag electrodes. Utilizes piezoelectricity for ultra-low force detection in bending mode [89]. Copyright © 2018 American Chemical Society. (**c**) Khan et al.: screen-printed P(VDF-TrFE) and MWCNT-PDMS composites. P(VDF-TrFE) for dynamic piezoelectric sensing; MWCNT-PDMS for static piezoresistive detection [90]. Copyright © 2014 IEEE. (**d**) Xiao et al.: screen-printed PVC/CB composites. Microcracks induced by cyclic bending enhance piezoresistive sensitivity [91]. Copyright © 2020 IOP Publishing Ltd. (**e**) Emon and Choi: screen-printed IL-MWNT composites in 3D-printed tires. Ionic conductivity and crack propagation enable pressure detection in automotive applications [92]. Copyright © 2017 by the authors. Licensee MDPI, Basel, Switzerland.

Polymer-based inks have revolutionized flexible sensor technology through their tunable properties and compatibility with scalable manufacturing. Below is a comprehensive review of key advancements emphasizing ink compositions, operational mechanisms, and technological applications:

A comparative investigation into multi-parametric sensing on paper substrates was conducted by Barmpakos et al., who evaluated inkjet-printed silver nanoparticle (Ag-NP) and PEDOT:PSS inks (Figure 5a). Ag-NPs demonstrated superior stability and humidity resistance due to their high conductivity, while PEDOT:PSS exhibited piezoresistive behavior but suffered from mechanical fragility. This work highlighted the critical role of ink–substrate interactions in sensor performance.

Printable poly(vinylidenefluoride-co-trifluoroethylene) (P(VDF-TrFE)) ink for piezoelectric sensors was developed by Rajala et al. through solution processing (Figure 5b). The ink, paired with vacuum-evaporated silver electrodes, enabled direct poling without stretching, achieving high piezoelectric coefficients (d_33_ up to 25 pC/N). However, recent studies indicate that the d_33_ value of P(VDF-TrFE) films can vary significantly depending on processing parameters. In contrast, typical solution-processed films exhibit d_33_ values in the range of 20–40 pC/N [87]. These advancements highlight the potential of P(VDF-TrFE) ink-based sensors for high-sensitivity applications, particularly in biomechanical and flexible electronics domains.

Screen-printed tactile sensors incorporating both piezoelectric and piezoresistive mechanisms were reported by Khan et al. P(VDF-TrFE) ink required multi-step assembly for dynamic force detection, while MWCNT-PDMS composites offered simpler static sensing through piezoresistive pathways (Figure 5c). This study underscored the trade-offs between different sensing modalities in conformable systems.

Crack-enhanced strain sensors were fabricated by Xiao et al. using screen-printed polyvinyl chloride/carbon black (PVC/CB) composites (Figure 5d). Cyclic bending induced microcracks that amplified piezoresistive responses, achieving gauge factors up to 1563. This work demonstrated the potential of crack engineering in polymer-based sensors.

A piezoresistive sensor embedded in 3D-printed tires was presented by Emon and Choi, utilizing screen-printed ionic liquid (IL)-MWNT composites (Figure 5e). The sensor detected dynamic forces via ionic conductivity and crack propagation, showcasing innovative integration with automotive structures.

### 4.4. Composite Inks

**Figure 6:**
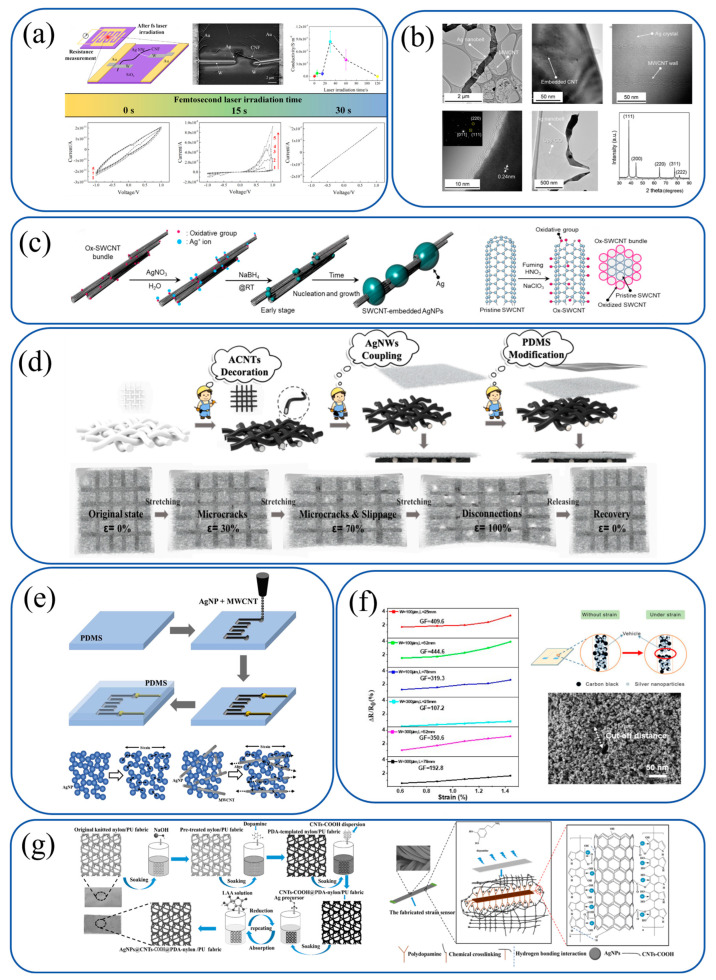
Composite ink systems for flexible sensor applications. (**a**) Feng et al.: carbon nanofibers (CNFs) and silver nanowires (AgNWs). CNFs provide mechanical robustness; AgNWs enable laser-induced bonding via plasmonic energy transfer [93]. Copyright © 2021 Elsevier Ltd. (**b**) Han et al.: UPy-modified nanocarbons (MWCNTs/GO) and Ag nanobelts. UPy groups template Ag nanobelt growth; nanocarbons enhance conductivity and stability [94]. Copyright © 2017 The Author(s). (**c**) Lee et al.: oxidized SWCNTs and AgNPs. Ox-SWCNTs act as nucleation templates for AgNP growth, forming pearl-necklace-like structures [95]. Copyright © 2021 The Author(s). (**d**) Lin et al.: acid-modified carbon nanotubes (ACNTs), AgNWs, and PDMS. ACNTs form internal conductive networks; AgNWs create external layers for high sensitivity [96]. Copyright © 2021 Elsevier B.V. (**e**) Min et al.: AgNP-MWCNT composites via AFN printing. MWCNTs provide stretchability; AgNPs enhance tunneling sensitivity through percolation paths [97]. Copyright © 2018 Elsevier Ltd. (**f**) Qi et al.: carbon black (CB)/AgNP composites. CB improves crack propagation; AgNPs enhance conductivity for strain sensing [98]. Copyright © 2020 MDPI. (**g**) Zhao et al.: CNTs-COOH/AgNP composites on PDA-templated fabrics. PDA promotes adhesion; CNTs-COOH/AgNPs form conductive networks with electrothermal properties [99]. Copyright © 2021 Elsevier B.V.

Composite ink systems combining metallic and carbon-based nanomaterials have unlocked new frontiers in flexible sensor technology, leveraging synergistic properties to address challenges in conductivity, stretchability, and durability. Below is a comprehensive review of key advancements, emphasizing material design, operational mechanisms, and technological applications:

A hybrid nanomaterial system integrating carbon nanofibers (CNFs) and silver nanowires (AgNWs) was developed by Feng et al., who demonstrated femtosecond laser-induced bonding for flexible electronics (Figure 6a). CNFs, acting as robust conductive scaffolds, were paired with PVP-functionalized AgNWs to enable plasmonic energy transfer. Optimized laser parameters (7.0–10.0 mJ/cm^2^ fluence) reduced interfacial resistance by six orders of magnitude, creating networks with ultra-high sensitivity.

Bio-inspired Ag/nanocarbon hybrids were introduced by Han et al., who utilized UPy-modified nanocarbons (MWCNTs/GO) as templates for Ag nanobelt synthesis (Figure 6b). UPy groups promoted anisotropic growth of Ag nanobelts, while nanocarbons provided mechanical stability and percolation pathways. This additive-free approach yielded conductive materials with ≥1000 S/cm conductivity.

Lee et al. reported AgNP-SWCNT composites fabricated using oxidized SWCNTs as nucleation templates (Figure 6c). Chlorate-based oxidation functionalized SWCNTs, enabling room-temperature AgNP growth. Screen-printed films with Ag flakes and PDMS exhibited 4907 S/cm conductivity and >10,000-cycle durability under 20% strain.

A multi-layered TPU-based nanofiber sensor was presented by Lin et al., integrating acid-modified carbon nanotubes (ACNTs) and self-assembled AgNWs (Figure 6d). ACNTs formed internal conductive networks, while AgNWs created external layers. PDMS coating conferred superhydrophobicity, achieving high sensitivity through hierarchical conductive structures.

Stretchable AgNP-MWCNT sensors were developed by Min et al. via aerodynamically focused nanomaterial (AFN) printing (Figure 6e). Adjusting AgNP:MWCNT ratios balanced sensitivity and stretchability. AFN printing ensured robust adhesion, leveraging MWCNTs for flexibility and AgNPs for tunneling sensitivity.

Screen-printed CB/AgNP composites were optimized by Qi et al., achieving high sensitivity through rheological tuning and pattern optimization (Figure 6f). This composite ink demonstrated stability across temperature/humidity variations, highlighting its potential for wearable applications.

CNTs-COOH/AgNP composites on PDA-templated fabrics were fabricated by Zhao et al. via dip-coating (Figure 6g). PDA enhanced adhesion through hydrogen bonding, while AgNPs grew via metal chelation. This system enabled conductive networks with electrothermal functionality and a wide strain sensing range.

The core of the printed electronics manufacturing process is obtaining colloidal solutions with specific rheological properties. Various materials have been developed and applied to achieve low-cost, high-resolution, ultra-stable, and diverse printed electronics. The host materials of printed, electronic inks are functional materials with various functions, and the essential components of the ink system include active elements, binders, solvents, and additives [100,101]. The proportion of solvent or dispersant in ink is the largest and choosing a suitable solvent or dispersant will obtain an excellent printing effect. In addition, printed electronic inks, solvents, or dispersants can also directly affect the functionality of the resulting film and, thus, the performance of the entire electronic device. To obtain good printing results and performance indicators, printed electronic inks also contain various additives, such as wetting agents, defoaming agents, and adhesion promoters [102,103]. The functions of additives are different. Some can adjust the viscosity and surface tension of the ink, some can adjust the drying speed of the ink, and some can improve the stability of the ink and the uniformity of dispersion. Printed electronic inks are ultimately deposited on the surface of various materials to form thin films, so the adhesion of inks on multiple substrates and materials is the primary issue. In addition to considering the characteristics of the ink itself, various treatments can be performed on the surface of the carrier to increase the adhesion of the ink on the surface [104,105]. Whether the surface tension of printed electronic ink is controlled correctly will directly affect the printing effect. First of all, the most important thing is the ink leveling on the substrate. The substance with low surface tension is easy to spread on the surface of the essence with high surface tension. The adhesion on the substrate, the higher the surface tension of the ink, the worse the wetting of the substrate, and the reduction in the bonding point between the primary material and the substrate, thereby affecting the adhesion. In addition, when the surface tension of the substrate is low or the surface tension of the ink is high, the printed film will appear to shrinkage and other phenomena [106,107]. The solvent and additives mainly determine the viscosity of the ink. Appropriate viscosity thickness achieves a good inkjet effect, ensuring high-speed printing and reducing the risk of plugging [108]. Depending on the function performed by the printed electronic ink, the drying speed required is also different. In adjusting the drying speed of the ink and controlling the temperature, humidity, wind speed, and other conditions, improvements are mainly caused by the ink itself due to factors such as the choice of the boiling point of the solvent, the concentration of the ink, and so on. While materials for flexible electronics are becoming smaller, more robust, lighter, cheaper, and more durable, it is critical to consider their impact on human health and the environment. Therefore, addressing biocompatibility, toxicity, and environmental risks during processing and post-use degradation will help standardize their use in wearables, e-textiles, and personalized medical devices.

When selecting substrate materials for printing, it is essential to consider their specific mechanical properties. We have summarized the mechanical properties of common materials, which can be referenced in Table 1. For instance, highly rigid substrates such as metals and ceramics may necessitate inks with different viscosity levels compared to softer substrates like fabric or paper. The ink’s viscosity must be optimized to ensure proper adhesion and coverage on each substrate type. The temperature characteristics of the substrate also significantly influence the printing process. Some substrates, such as glass and ceramics, exhibit higher heat resistance, permitting higher curing temperatures during printing. Conversely, substrates with lower heat resistance (e.g., plastics or certain fabrics) necessitate lower curing temperatures to prevent deformation or damage. A summary of common mechanical properties of materials is presented in Table 2. The precision and success of printing largely depend on understanding the unique properties of each substrate and selecting compatible ink formulations accordingly. Neglecting the substrate-material interaction can lead to issues such as poor ink adhesion, smearing, or color distortion. Hence, researchers and practitioners in the field of printing must conduct thorough research and adopt appropriate printing parameters based on the material type to achieve optimal results. [46,109,110,111,112,113,114,115,116].

## 5. Characteristics of Inks and Substrates

The key to ensuring the output quality of printed electronics depends on the viscosity of the ink, surface tension, and the physical properties of the substrate characterizing the ink [117].

Rheology is the study of the response of substances (mainly fluids) to force and deformation. The mechanical (mechanical) responses of substances are divided into elastic deformation, viscous flow, and combinations. Rheological measurements and their analysis are crucial for controlling the fluidity of meanings [118]. Information on the behavior of fluids under stress is vital for ink adaptation and final resolution in different printing processes and the quality of deposited films. Fluids are usually divided into Newtonian fluids and non-Newtonian fluids. Newtonian fluids can be characterized by a single viscosity coefficient at a specific temperature. The viscosity of such fluids varies with temperature but not with strain rate [119]. Newtonian fluids exhibit constant thickness at a constant temperature. Fluids whose density varies with strain rate (relative flow rate) are called non-Newtonian fluids, shared in our lives, and fluids for printing electronic inks.

All printing and coating technologies require different compositions and viscosities of inks [120]. The viscosity of inks is a rheological property that is central to achieving high-quality printable films. It affects ink availability, transfer, final film functionality, printed pattern resolution, and high aspect ratio. Good printing quality can only be achieved when the viscosity of the ink is adapted to the specific printing technology [121]. Only when the thickness of the ink is compatible with the selected technology will the ink be printable, and the correct film will be printed. Regarding rheology, different requirements must be met for specific printing technology. The basic needs of standard printing technologies for ink viscosity are shown in Table 3.

Shear rate: $\dot{\gamma }$ = v/h with shear rate $\dot{\gamma }$ (pronounced: gamma dot), velocity v (in m/s), and shear gap h (in m). The unit for shear rate is 1/s = 1 s^−1^, also called reciprocal seconds [124,125].

Shear stress: τ = F/A with shear stress τ (pronounced: tau), shear force F (in N, Newton), and shear area A (in m^2^). The unit for shear stress is 1 N/m^2^ = 1 Pa (Pascal). A rheometer records the sheer force via the torque at each measuring point [124,125].

Viscosity: with viscosity η (pronounced: eta), shear stress τ (in Pa), and shear rate $\dot{\gamma }$ (in s^−1^) [124,125].

### 5.1. Surface Tension

Surface tension is a property of a fluid interface where molecules on the surface of a liquid encounter fewer molecules that can interact with each other than inside the bulk phase. So, from an energy point of view, there is less benefit to being present at the surface [126,127]. Liquids are assumed to have a minimal surface area without external forces. Surface tension is the work required to interfere with a fluid’s droplet shape with intermolecular forces. These intermolecular forces hold the fluid together at any air or liquid interface. The surface tension in ink depends on its solvent, and tiny amounts of polar or non-polar surfactants can alter the surface tension [128,129]. Surface tension determines the behavior of liquids in many processes and phenomena: wetting and wettability, droplet size, and dispersion and dispersibility. The degree of wetting of solids by drinks depends on surface tension for several reasons, which is also very important in the wetting process of printing and spraying. There are several methods to measure surface tension in liquids; the rod method, like the plate method, uses a cylindrical rod with a small wetted length to measure small volumes of liquids. The bubble pressure method measures the maximum internal pressure of a bubble formed in a drink through a capillary. Drop Volume Method: The importance of a drop of liquid produced at a vertical vein is measured as it detaches. This method is mainly used to measure interfacial tension [130]. Usually, the pendant drop method is used, and the droplet’s shape hanging from the needle is determined by the droplet’s surface tension and weight. Surface tension can be selected from droplet images using droplet shape analysis.

### 5.2. Contact Angle and Surface Energy

The contact angle is usually defined as the interface between a liquid and a solid when they are in contact; the angle between the liquid surface and the contact surface profile is called the contact angle θ (lowercase letters). Contact angle (wetting angle) is a measure of the wetting of a solid by a liquid. Contact angle measurements provide information about the ink-surface interaction (the behavior of ink droplets on the surface of the substrate) and their wetting behavior [131,132].

The contact angle is 0° under complete wetting. Solids are wettable between 0° and 90°, and solids above 90° are not wettable. The contact angle approaches the limit of 180° in the case of superhydrophobic materials. Temperatures such as surface roughness, ink formulation, solvent properties, surface energy, pre- and post-treatment (UV, plasma), and treatment temperature can all affect the contact angle. Having a certain wettability is a requirement for printability [133,134].

The surface energy of a solid material (substrate) is measured and defined as the number of intermolecular forces available on the material’s surface. These forces can be exerted on the fluid droplet to break its interfacial bonds. Usually, the effect of bonding occurs between the interfaces that are in contact with each other. The bonding strength mainly depends on the wettability (the uniform spreading of the liquid under the action of the intermolecular force on the solid surface/the affinity of the liquid for the stable) and the adhesive force (the formation of sufficient adhesion between the adhesive and the adherend) force) are influenced by both. The wettability is mainly determined by the surface energy of the bond and the adherend. (The liquid is called surface tension, and the solid is called surface energy.) The bonding ability can be known from such a principle. Techniques such as corona, UV–ozone, sintering, plasma, and laser treatments alter the substrate’s surface energy. The controllability of the surface energy is crucial for printed electronics [135,136,137].

## 6. Applications and Challenges of Printing Flexible Strain Sensors

A flexible sensor refers to a sensor made of flexible materials with good flexibility and elasticity and can even be bent or folded freely. It has flexible and diverse structures and can be arbitrarily arranged according to the requirements of measurement conditions. Measure to detect. New flexible sensors have been widely used in electronic skin, healthcare, electronics, electrical engineering, sports equipment, textiles, aerospace, environmental monitoring, etc. [138]. The flexible strain sensor is a critical element in monitoring deformation. Conventional metal- or semiconductor-based strain gauges cannot meet the basic requirements of conformal fit to the surface of the human body or flexible devices, and the sensing range is smaller than practical. Most of the existing elastic strain sensors are based on the contact resistance mechanism, that is, through various sensing materials and the corresponding microstructure design to realize the change in the contact relationship of the conductive microstructure (the transition from contact to separation, or the distance relationship involving the seepage effect and the tunnel effect), thereby forming the stretchability and resistance changes in the sensor. Such elastic strain sensors are commonly prepared from carbon-based materials (including carbon nanotubes, graphene, carbonized silk, carbon black, etc.) or metal nanowires and nanoparticles [139,140,141,142]. The corresponding microstructures include spring-like structures, island gap structures, and buckling sheath cores. Fiber structure and fish scale-like structure, etc. These strain sensors usually have an extensive sensing range and sensitivity coefficient. Still, the repeatability and linearity of their electrical responses need to be improved because their core sensing mechanism involves an unstable contact relationship: complex slippage on the contact surface, friction, and adhesion relationships; nonlinear deformation of microstructures; transformation of contact modes [143,144,145].

Table S1 summarizes the key performance metrics of various flexible strain sensors, including the printing technologies used, the resolution or line width achievable, and the applications across different fields. These data provide a detailed comparison of materials and printing methods, highlighting the performance characteristics and challenges for each sensor type.

Flexible strain sensors have emerged as a key technology in the field of printed electronics due to their potential to enable advanced applications in various sectors, including healthcare, robotics, wearable technology, and smart infrastructure. This section explores the diverse applications of these sensors enabled by printing technology, along with the challenges that need to be addressed for their widespread adoption.

### 6.1. Applications of Flexible Strain Sensors

#### 6.1.1. Wearable Devices for Health Monitoring

One of the most prominent applications of flexible strain sensors is in the realm of wearable health monitoring devices. These sensors are capable of detecting subtle deformations in the skin, making them ideal for monitoring physiological signals such as pulse, respiration rate, and muscle activity. By integrating strain sensors into flexible substrates that can conform to the human body, wearable devices provide non-invasive monitoring of vital signs, which is crucial for personalized healthcare [146,147,148,149].

Printed flexible strain sensors offer the advantage of low-cost, scalable manufacturing, which is especially important for consumer products that require mass production. The sensors’ flexibility ensures user comfort during prolonged use, making them suitable for continuous monitoring applications like fitness tracking and chronic disease management.

#### 6.1.2. Human–Machine Interfaces (HMI)

In the context of human–machine interfaces, flexible strain sensors play a vital role in developing responsive and intuitive systems. These sensors can be used to create gesture recognition devices that translate physical movements into electronic signals, allowing users to interact with machines through touch, bending, or pressure. Applications include virtual reality (VR) gloves and smart textiles that enable the control of electronic devices through gestures.

The integration of printed strain sensors into soft robotics is another significant area of application. Soft robots equipped with these sensors can perform complex movements while being sensitive to external forces, which is essential for delicate manipulation tasks in industries such as food handling and healthcare [150,151,152,153].

#### 6.1.3. Structural Health Monitoring

Flexible strain sensors are also increasingly used in structural health monitoring (SHM) systems for buildings, bridges, and other infrastructure. By embedding or attaching strain sensors to critical structures, it is possible to monitor deformations, stress, and potential damage in real time, thereby ensuring the safety and longevity of these assets [154,155].

Printed sensors are advantageous in this domain due to their lightweight nature and ease of deployment over large surfaces. They can be applied in situ using printing techniques, which reduces installation costs and enables continuous, real-time monitoring without significant structural modifications. For example, sensors printed on flexible substrates can be used to monitor crack propagation in concrete or strain distribution in bridges during heavy loads.

#### 6.1.4. E-Skin and Prosthetics

Electronic skin (e-skin) is another emerging application that relies on flexible strain sensors. E-skin mimics the functionality of human skin by detecting mechanical stimuli like pressure and strain, making it suitable for integration into prosthetic limbs. It provides sensory feedback to users, allowing them to perceive pressure and touch through their artificial limbs.

The printed nature of these sensors allows for large-area production and customization to fit various shapes and sizes, enhancing their adaptability to different prosthetic devices. The sensitivity and flexibility of the printed sensors are crucial for creating a natural touch sensation improving the user experience [156,157,158].

#### 6.1.5. Internet of Things (IoT) and Smart Textiles

With the rise of the Internet of Things (IoT), flexible strain sensors are being integrated into smart textiles and connected devices, allowing for real-time data transmission and monitoring. These sensors can be embedded into fabrics to create smart garments that monitor body movement or detect external environmental changes like pressure or impact.

Printed electronics provide a practical solution for integrating sensors into textiles without compromising the fabric’s flexibility and comfort. This opens up opportunities for applications in sportswear, safety gear, and even smart furniture, where continuous monitoring and data logging are desired [159,160,161,162].

### 6.2. Challenges in the Development of Printed Flexible Strain Sensors

#### 6.2.1. Material Development and Conductive Inks

The development of suitable conductive inks is one of the primary challenges in advancing printed strain sensors. Conductive inks are essential for forming the active sensing layers, but achieving a balance between conductivity, flexibility, and adhesion to substrates is challenging. Materials like silver nanowires, carbon nanotubes, and conductive polymers are often used, but each has limitations in terms of cost, processability, and environmental stability. Ink formulation must ensure that the printed patterns maintain their integrity during deformation, which requires precise control over ink viscosity, particle size, and surface tension [163,164].

#### 6.2.2. Printability and Process Optimization

Achieving high-resolution, consistent, and repeatable patterns is crucial for the performance of printed sensors. Factors like printing speed, substrate temperature, and ink deposition thickness must be carefully optimized. Different printing techniques, such as screen printing, inkjet printing, and gravure printing, offer distinct advantages but also come with challenges, such as nozzle clogging in inkjet printing or resolution limitations in screen printing. Printability also affects the uniformity of the sensor’s conductive networks, which directly impacts their sensitivity and durability [165,166]. Another major challenge lies in ensuring strong adhesion between printed inks and substrates. Poor adhesion can lead to delamination, cracking, and degradation of printed patterns over time, particularly under repeated mechanical deformation. Surface treatment techniques, such as plasma treatment, corona treatment, and chemical etching, have been employed to enhance ink–substrate interactions. However, optimizing these treatments for different materials remains complex, as each substrate responds differently to surface modifications. For instance, plasma and corona treatments are highly effective for increasing the surface energy of polymeric substrates but may require fine-tuning to avoid unwanted alterations in mechanical properties. Similarly, chemical etching can improve adhesion by creating micro-roughened textures but may introduce material compatibility issues or require additional processing steps. Balancing these surface treatment methods with scalable and cost-effective sensor manufacturing remains an ongoing challenge. Furthermore, achieving printability at an industrial scale while maintaining sensor performance introduces trade-offs between resolution, material selection, and processing efficiency. Ink formulation must be optimized to prevent drying-related issues during high-speed printing while ensuring compatibility with diverse substrates. Additionally, environmental factors such as humidity and temperature fluctuations during printing can lead to inconsistencies in ink deposition, further complicating reproducibility. Addressing these challenges requires continuous advancements in materials science, printing technology, and process control to enhance the scalability, reliability, and functionality of printed flexible strain sensors.

#### 6.2.3. Substrate Compatibility and Flexibility

Substrate choice plays a critical role in the flexibility and durability of printed strain sensors. Flexible polymers like polyimide, polyethylene terephthalate, and thermoplastic polyurethane are commonly used, but each has trade-offs in terms of thermal stability, mechanical strength, and surface energy, which affect ink adhesion and overall sensor performance. Achieving good adhesion between the ink and the substrate without compromising the flexibility of the entire device is a significant challenge [148,167].

#### 6.2.4. Sensitivity and Long-Term Stability

For practical applications, strain sensors need to exhibit high sensitivity and long-term stability under repeated mechanical deformations. However, maintaining performance over thousands or even millions of cycles is challenging, as repeated bending and stretching can lead to microcracks in the conductive patterns, degrading the sensor’s signal [166,168].

#### 6.2.5. Cost and Scalability

While printing technology is generally considered cost-effective for large-scale production, the initial costs associated with developing specialized inks and optimizing the printing processes can be high. The commercialization of printed flexible strain sensors is heavily influenced by cost and scalability, which are crucial for large-scale applications like wearables and health monitoring. Several factors contribute to the overall cost, including material selection, manufacturing techniques, and production volume. The choice of materials significantly impacts costs. While silver nanoparticle-based inks offer high conductivity, they are expensive, and more affordable alternatives like carbon-based materials (e.g., graphene, CNTs) and conductive polymers (e.g., PEDOT:PSS) offer lower-cost options with sufficient performance. Substrate costs also vary, with polymer films and paper-based materials being more economical compared to high-performance alternatives. Scalability depends on the printing method. Inkjet printing is ideal for prototyping but may struggle with high-throughput production, while screen printing and gravure printing enable faster production but may require adjustments for fine resolution. Roll-to-roll (R2R) manufacturing offers the best scalability for large-volume production, making it a key approach for cost-effective, high-throughput sensor fabrication. Optimizing printing processes can also help reduce costs, such as through ink formulation, curing optimization, and hybrid printing methods (combining additive and subtractive techniques) to minimize material waste and energy consumption. Low-temperature sintering and water-based inks further contribute to cost reductions and environmental benefits. For printed sensors to compete with traditional silicon-based sensors, they must be cost-effective, reliable, and stable. While printed electronics offer flexibility and compatibility with large-area manufacturing, addressing challenges like device variability and long-term stability is crucial for broader adoption, particularly in disposable and wearable applications. To ensure widespread adoption, research should focus on developing lower-cost conductive materials, enhancing printing precision, and improving automated R2R manufacturing processes. Collaboration between academia and industry will be essential for transitioning from lab-scale to commercial production, enabling cost-effective and high-performance printed flexible strain sensors. [169,170].

#### 6.2.6. Degradation Mechanisms and Sustainable Strategies

The environmental sustainability of printed flexible strain sensors is a growing concern due to the use of non-biodegradable materials and the accumulation of electronic waste. Addressing this challenge requires integrating biodegradable substrates (e.g., cellulose, silk fibroin, PLA) and recyclable conductive materials (e.g., graphene, CNTs, transient metals like Mg and Zn) to reduce environmental impact. Additionally, eco-friendly fabrication processes, such as low-waste additive printing and solvent-assisted material recovery, can enhance recyclability. Future advancements should focus on improving the stability of biodegradable inks, optimizing transient materials, and developing scalable recycling strategies to align with circular economy principles. By combining sustainable materials with innovative manufacturing techniques, printed strain sensors can achieve high performance while minimizing their ecological footprint.

## 7. Conclusions

The review of flexible strain sensors based on printing technology: conductive ink development, multiple substrates, printability, and application highlights the significant progress and potential of printed flexible strain sensors in advancing various technological fields, including wearable devices, human–machine interfaces, and structural health monitoring. Through the development of innovative conductive inks, optimization of substrates, and improvements in printability, these sensors offer a promising solution for creating lightweight, low-cost, and versatile sensing systems.

One of the major findings is that the success of these sensors relies heavily on the appropriate choice and formulation of conductive inks, which directly impacts the sensitivity, flexibility, and durability of the sensors. Additionally, selecting suitable substrates that are compatible with different printing techniques plays a crucial role in achieving high-performance, mechanically robust devices. The ability to optimize the printing process is key to achieving uniformity and precision in sensor fabrication, enabling better control over the sensor’s mechanical and electrical properties.

Despite these advances, the field faces several challenges, such as ensuring long-term stability, reproducibility, and scalability for industrial applications. Addressing these issues will require a combination of material innovations, improved printing techniques, and better integration strategies. Further research should focus on developing more environmentally friendly conductive inks and exploring novel substrate materials that could enhance the overall performance of printed strain sensors.

Overall, this review underscores the tremendous potential of printing technology in developing flexible strain sensors, positioning them as crucial components for next-generation sensing applications. With continued advancements and interdisciplinary collaboration, printed flexible strain sensors can be expected to play a pivotal role in the growth of smart technologies, enabling new applications across various sectors while contributing to the evolution of flexible electronics.

## Figures and Tables

**Table 1:** Mechanical properties of common substrate materials.

Material (Abbreviation)	Tensile Strength (MPa)	Young’s Modulus (MPa)	Elongation at Break (%)	Density (g/cm^3^)
Polyimide (PI)	100–150	2500–4000	5–10	1.38–1.43
Polyethylene Terephthalate (PET)	50–70	2800–4000	50–300	1.38–1.40
Polyurethane (PU)	20–60	10–1000	100–800	1.0–1.2
Polydimethylsiloxane (PDMS)	4–10	0.5–10	100–800	1.0–1.1
Ecoflex	2–5	0.01–0.1	400–1000	1.0–1.1
Kapton (a type of PI film)	110–140	3000–3500	5–8	1.42
Nylon (Polyamide, PA)	40–90	1500–3000	10–300	1.14–1.15
Polypropylene (PP)	20–35	1000–1500	200–800	0.89–0.91
Polyvinyl Chloride (PVC)	40–60	2500–4000	2–40	1.3–1.5
Silk Fabric	20–40 (depending on type)	500–1000	15–30	1.3–1.4
Cotton Fabric	2–5 (per yarn strength)	100–300	3–10	1.5–1.6
Paper	10–30 (tensile strength of paper sheet)	100–500	1–5	0.6–1.2
Lycra (Spandex)	1–5 (initial modulus)	0.1–1	500–800	1.2–1.3
Polyester Fabric	40–70 (yarn strength)	1000–2000	15–30	1.3–1.4
Rubberized Fabric	10–30 (depending on rubber content)	50–200	100–500	1.1–1.3
Polyethylene (PE)	7–40	400–1500	10–1000	0.91–0.96
Polystyrene (PS)	35–50	2500–3500	1–3	1.04–1.07
Acrylonitrile Butadiene Styrene (ABS)	30–50	1800–2800	10–40	1.04–1.06
TPU (Thermoplastic Polyurethane)	20–60	10–1500	100–800	1.1–1.2

**Table 2:** Specifications of various substrates.

Material (Abbreviation) 000	Surface Roughness (nm)	Dielectric Constant	Thermal Expansion Coefficient (ppm/°C)	Electrical Resistivity (Ω·cm)	Optical Transmittance (%)	Chemical Stability
Polyimide (PI)	1–10	3–4	20–50	>10^15^	70–90 (for thin films)	Excellent against high temperature, most acids, alkalis, and organic solvents
Polyethylene Terephthalate (PET)	2–15	3–3.5	60–180	>10^14^	85–92	Good against many chemicals but attacked by strong alkalis and some hot solvents
Polyurethane (PU)	5–20	2.5–3.5	100–300	>10^13^	80–90 (clear grades)	Moderate against oils and abrasion, can be degraded by some chemicals
Polydimethylsiloxane (PDMS)	0.5–5	2.5–3	200–300	>10^15^	90–95	Good against water, oxygen, and many organic solvents
Ecoflex	1–10	2–3	300–500	>10^14^	80–90 (for thin films)	Resistant to many common chemicals, good flexibility
Nylon (Polyamide, PA)	3–15	3–4.5	70–120	>10^13^	80–90 (for some grades)	Good against oils, fuels, and abrasion, affected by strong acids and bases
Polypropylene (PP)	2–10	2.2–2.6	100–200	>10^15^	80–90 (for clear grades)	Resistant to most organic solvents and many chemicals
Polyvinyl Chloride (PVC)	3–15	3–4	50–100	>10^13^	75–85 (for clear grades)	Resistant to many acids, alkalis, and salts, softened by some solvents
Silk Fabric	5–20 (for Bombyx mori silk)	2–3	80–150	>10¹³	70–80 (for thin films)	Moderate against common chemicals, sensitive to strong alkalis
Cotton Fabric	10–30 (per yarn)	1.5–2.5	50–100	>10^12^	50–70 (for thin layers)	Susceptible to hydrolysis by strong acids and alkalis
Paper	10–50 (for common printing paper)	1.8–2.5	30–80	>10^12^	20–40 (for white paper)	Easily damaged by water and strong chemicals
Lycra (Spandex)	1–5 (for smooth surfaces)	2–3	200–400	>10^13^	70–80 (for thin films)	Resistant to most common chemicals in clothing applications
Polyester Fabric	5–15 (yarn surface)	3–3.5	60–120	>10¹³	80–90 (for some grades)	Good against most chemicals and abrasion
Rubberized Fabric (with natural rubber)	10–30 (depending on rubber content)	2.5–3.5	150–300	>10^13^	60–80 (for thin films)	Resistant to water and some abrasion, chemical resistance depends on rubber type
Polyethylene (PE)	1–8	2.3–2.5	150–300	>10^15^	80–90 (for clear grades)	Good against many organic solvents and weak acids/bases
Polystyrene (PS)	2–10	2.4–2.6	60–80	>10^14^	85–90	Poor against many organic solvents
Acrylonitrile Butadiene Styrene (ABS)	3–15	2.4–3.2	80–120	>10^13^	50–70 (for some grades)	Moderate against chemicals, attacked by some strong solvents
TPU (Thermoplastic Polyurethane)	5–20	2.8–3.5	100–200	>10^13^	80–90 (for clear grades)	Good abrasion and oil resistance, some sensitivity to polar solvents

**Table 3:** The basic needs of standard printing technologies for ink viscosity [122,123].

Printing Method	Printing Speed [m/min]	Minimum Resolution [μm]	Ink Viscosity [mPa·s]	Surface Tension [mN/m]	Advantages	Limitations
Screen Printing	0.1–15	50	500–50,000	35–50	Very low prototyping cost	Limited resolution
					Balance between speed, reliability, and cost	Strict ink rheology requirements
Gravure	1–2	30	10–200	20–40	Simple process	High startup costs
					High speed	Expensive prototyping
					Good reliability	
					Suitable for long production runs	
Inkjet	0.01–15	30	1–100	10–30	No additional prototyping cost	Difficulty integrating with roll-to-roll systems
					Excellent resolution and pattern control	Nozzle clogging affects reliability
Aerosol Jet	0.01–1	10	5–10,000	10–50	Compatible with non-planar surfaces	High susceptibility to clogging
					Rapid prototyping	Limitations in ink design
					Compatible with many materials	Low throughput
Electro-hydrodynamic	0.01–0.5	5	100–10,000	20–40	Excellent resolution	Low throughput
					Rapid prototyping	Difficulty achieving high deposition heights
Flexography	10–100	30–100	50–500	25–45	High-speed printing	Limited resolution
					Cost-effective for large batches	Ink formulation complexity
Offset	5–30	10–20	30,000–100,000	30–50	High precision for complex patterns	High equipment cost
					Suitable for large-area production	Requires specialized ink formulation
Imprint/stamp	0.1–2	5–20	50–500	20–40	Simple tooling	Limited to flat surfaces
					Good for replicating micro-/nano-patterns	Low throughput for large quantities

## Data Availability

No new data were created or analyzed in this study. Data sharing is not applicable to this article.

## Supplementary Materials

The following supporting information can be downloaded at https://www.mdpi.com/article/10.3390/ma18092113/s1, Table S1: Quantitative Comparison of Materials, Printing Methods, and Applications in Printed Strain Sensors [76,78,80,83,95,98,99,171,172,173].
