# Supplementary material for: Flexible Strain Sensors Based on Printing Technology: Conductive Inks, Substrates, Printability, and Applications
Source: Materials (Basel). 2025 May 4;18(9):2113. doi: 10.3390/ma18092113 (PMC12072868; doi:10.3390/ma18092113)
Supplement: Supplementary file 1 [file materials-18-02113-s001.zip › materials-3559208-supplementary.pdf]

Support information

Xue Qi[ab], Jingjing Luo[ab], Haipeng Liu[ab], Shuheng Fan[ab], Zhongqi Ren[ab], Peike Wang[ab], Suzhu Yu[ab*] and Jun Wei[abc*]

[a]*Shenzhen Key Laboratory of Flexible Printed Electronics Technology, Harbin Institute of Technology (Shenzhen), Shenzhen 518055, China*

[b]*School of Materials Science and Engineering, Harbin Institute of Technology (Shenzhen), Shenzhen 518055, China*

[c]*State Key Laboratory of Advanced Welding and Joining, Harbin Institute of Technology (Shenzhen), Shenzhen 518055, China*

*Corresponding author: Xue Qi, Suzhu Yu, Jun Wei, *E-mail*: qixue@hit.edu.cn, szyu@hit.edu.cn, junwei@hit.edu.cn

Table S1. Quantitative Comparison of Materials, Printing Methods, and Applications in Printed Strain Sensors

**Materials**	**Printing Technology**	**Resolution/Line Width**	**Key Performance Metrics**	**Applications**	Reference
Ag nanopartices, CNTs,PDMS	Screen printing	120 µm, 228 µm	Conductivity: 248 S/cm, Stretchability:800%,Cyclic reliability:5000 cycles at 100% strain	Strain sensors for human motion detection	1
Ag nanopartices, SWCNTs,PDMS	Screen printing	10 µm	Conductivity:4907 S/cm, Stretchability:10,000 cycles at 20% strain	Stretchable electrodes and strain sensors	2
Graphene,PDMS	Spin coating	25 mm x 5 mm	Gauge factor:223 at 3% strain	Wearable wireless musical instrument	3
Various nanomaterials, polymers	3D printing	Varies	Sensitivity,flexibility stretchability,repeatability	Wearable electronics, electronic skin	4
AgNPs-decorated CNTs,nylon/PU fabrics,polydopamine	Dip-coating,liquid-phase chemical adsorption reduction	Not specified	High sensitivity durability over 1000 cycles,electrical heating property	Human motion detection,temperature detection	5

Ag nanodendrite nks,nitrile rubber	Roll-to-roll gravure printing	354±4.6 μm(width), 212±6.6 μm (gap)	Gauge factor:1.99~2.24, measurable strain range:-386×10^-6~16272×10^-6	Wearable electronics, human-machine interaction	6
Carbon black/Ag nanocomposites	Screen printing	Not specified	Gauge factor:444.5, durability:1000 cycles, linearity: R^2=0.9974	Wearable electronics, sports,health monitoring	7
Graphene-based inks	Screen printing, ultrafast aser direct writing	180 μm (electrode width),220 μm (spacing)	Gauge factor:550.14 (6-mm grid length)	Sensing deformation	8
Ag nanodendrite inks,SIS gel	Screen printing	2 mm (linewidth)	Gauge factor: 294.3, response time: 18 ms, stability:>3 weeks	Wearable electronics, human motion monitoring	9
Graphene aerogels	3D printing	100 μm (layer thickness)	Not specified	Composite materials, energy storage, stress sensor, thermal insulator	10

Reference

1. Huang, H.-J.; Ning, X.; Zhou, M.-B.; Sun, T.; Wu, X.; Zhang, X.-P. A Three-Dimensional Printable Liquid Metal-Like Ag Nanoparticle Ink for Making a Super-Stretchable and Highly Cyclic Durable Strain Sensor. ACS Appl. Mater. Interfaces 2021, 13, 18021–18032. https://doi.org/10.1021/acsami.1c01551.
2. Lee, J.-W.; Cho, J.Y.; Kim, M.J.; Kim, J.H.; Park, J.H.; Jeong, S.Y.; Seo, S.H.; Lee, G.-W.; Jeong, H.J.; Han, J.T. Synthesis of Silver Nanoparticles Embedded with Single-Walled Carbon Nanotubes for Printable Elastic Electrodes and Sensors with High Stability. Sci. Rep. 2017, 7, 10.1038/s41598-017-12307-5.
3. Liu, X.; Tang, C.; Du, X.; Xiong, S.; Xi, S.; Liu, Y.; Shen, X.; Zheng, Q.; Wang, Z.; Wu, Y.; Horner, A.; Kim, J.-K. A Highly Sensitive Graphene Woven Fabric Strain Sensor for Wearable Wireless Musical Instrument. Mater. Horiz. 2017, 4, 10.1039/C7MH00104E.
4. Liu, C.; Huang, N.; Xu, F.; Tong, J.; Chen, Z.; Gui, X.; Fu, Y.; Lao, C. 3D Printing Technologies for Flexible Tactile Sensors toward Wearable Electronics and Electronic Skin. Materials 2018, 11, 10.3390/ma11060943.
5. Zhao, S.-Q.; Zheng, P.-X.; Cong, H.-L.; Wan, A.-L. Facile Fabrication of Flexible Strain Sensors with AgNPs-Decorated CNTs Based on Nylon/PU

Fabrics through Polydopamine Templates. Applied Surface Science 2021, 536, 147824. https://doi.org/10.1016/j.apsusc.2020.147824.
6. Park, J.; Nam, D.; Park, S.; Lee, D. Fabrication of Flexible Strain Sensors via Roll-to-Roll Gravure Printing of Silver Ink. Smart Materials and Structures 2018, 27, 105013. https://doi.org/10.1088/1361-665X/aacbb8.
7. Qi, X.; Ha, H.; Hwang, B.; Lim, S. Printability of the Screen-Printed Strain Sensor with Carbon Black/Silver Paste for Sensitive Wearable Electronics. Materials 2020, 13, 4426. https://doi.org/10.3390/ma13194426.
8. Tseng, S.-F.; Liao, C.-H.; Hsiao, W.-T.; Chang, T.-L. Ultrafast Laser Direct Writing of Screen-Printed Graphene-Based Strain Electrodes for Sensing Glass Deformation. Ceramics International 2021, 47, 1043–1050. https://doi.org/10.1016/j.ceramint.2020.08.241.
9. Tian, B.; Yao, W.; Zeng, P.; Li, X.; Wang, H.; Liu, L.; Feng, Y.; Luo, C.; Wu, W. All-Printed, Low-Cost, Tunable Sensing Range Strain Sensors Based on Ag Nanodendrite Conductive Inks for Wearable Electronics. Journal of Materials Chemistry C 2018, 6, 12890–12896. https://doi.org/10.1039/C8TC04753G.
10. Zhang, Q.; Zhang, F.; Medarametla, S.P.; Li, H.; Zhou, C.; Lin, D. 3D Printing of Graphene Aerogels. Small 2016, 12, 1702–1708. https://doi.org/10.1002/smll.201503524
